# Inhibition of formyl peptide receptors improves the outcome in a mouse model of Alzheimer disease

**DOI:** 10.1186/s12974-020-01816-2

**Published:** 2020-04-24

**Authors:** Nicole Schröder, Anja Schaffrath, Josua A. Welter, Tim Putzka, Angelika Griep, Patrick Ziegler, Elisa Brandt, Sebastian Samer, Michael T. Heneka, Hannes Kaddatz, Jiangshan Zhan, Eugenia Kipp, Thomas Pufe, Simone C. Tauber, Markus Kipp, Lars-Ove Brandenburg

**Affiliations:** 1 Institute of Anatomy, Rostock University Medical Center, Gertrudenstrasse 9, D-18057 Rostock, Germany; 2 Department of Anatomy and Cell Biology, RWTH Aachen University, Aachen, Germany; 3 Department of Neurodegenerative Diseases and Gerontopsychiatry, University of Bonn, Bonn, Germany; 4 Institute for Occupational and Social Medicine, RWTH Aachen University, Aachen, Germany; 5 German Center for Neurodegenerative Diseases (DZNE), Bonn, Germany; 6 Department of Neurology, RWTH University Hospital Aachen, Aachen, Germany

**Keywords:** Alzheimer disease, Amyloid beta, Formyl peptide receptor, Glia cell, Microglia, Innate immunity, Annexin A1

## Abstract

**Background:**

An important hallmark of Alzheimer’s disease (AD) is the increase of Aβ1-42 burden and its accumulation to senile plaques, leading the reactive gliosis and neurodegeneration. The modulation of glia cell function represents an attractive therapeutic strategy, but is currently limited by an incomplete understanding of its relevance for AD. The chemotactic G-protein coupled formyl peptide receptor (FPR), which is known to modulate Aβ1-42 uptake and signal transduction, might be one candidate molecule regulating glia function in AD. Here, we investigate whether the modulation of FPR exerts beneficial effects in an AD preclinical model.

**Methods:**

To address this question, APP/PS1 double-transgenic AD mice were treated for 20 weeks with either the pro-inflammatory FPR agonist fMLF, the FPR1/2 antagonist Boc2 or the anti-inflammatory FPR2 agonist Ac2-26. Spatial learning and memory were evaluated using a Morris water maze test. Immunohistological staining, gene expression studies, and flow cytometry analyses were performed to study neuronal loss, gliosis, and Aß-load in the hippocampus and cortex, respectively.

**Results:**

FPR antagonism by Boc2-treatment significantly improved spatial memory performance, reduced neuronal pathology, induced the expression of homeostatic growth factors, and ameliorated microglia, but not astrocyte, reactivity. Furthermore, the elevated levels of amyloid plaques in the hippocampus were reduced by Boc2-treatment, presumably by an induction of amyloid degradation.

**Conclusions:**

We suggest that the modulation of FPR signaling cascades might be considered as a promising therapeutic approach for alleviating the cognitive deficits associated with early AD. Additional studies are now needed to address the downstream effectors as well as the safety profile of Boc2.

## Background

On the histopathological level, senile plaques and neurofibrillary tangles characterize Alzheimer’s disease (AD). An important component of the AD plaque is the accumulation of amyloid-β 1–42 (Aβ1-42), a 42 amino acid peptide fragment derived from sequential proteolytic cleavage of the amyloid precursor protein by beta- and gamma-secretase enzymatic activities [1]. Aβ1-42 plays a central role during the activation of glia cells (i.e., astrocytes and microglia) and the consequent release of proinflammatory cytokines, chemokines, or reactive oxygen species, eventually leading to neurodegeneration [2].

The precise role of glial cells, in particular astrocytes and microglia, in AD remains speculative. On the histological level, glial cells are closely associated with amyloid plaques and show a reactive phenotype [3]. In general, astrocytes, in concert with microglia, are key regulators of the brain’s inflammatory response. The activation of astrocytes, characterized by cellular hypertrophy and an increase in glial fibrillary acidic protein (GFAP) expression, is a histopathological hallmark of diverse neurological disorders, including AD. Moreover, the degree of astrogliosis is correlated with the extent of cognitive decline in AD and in AD models [4, 5]. Microglia cells, considered as the resident innate immune cells of the central nervous system (CNS), are critically involved in pathogen recognition and are considered to represent the innate immune system of the CNS [6]. In AD pathogenesis, microglia activation may play a dual role: on one side, acute microglial activation in some experimental paradigms leads to decreased Aβ accumulation by increasing its phagocytosis or clearance, pointing towards a protective function of microglia cells in AD. In contrast, the chronic activation of microglia contributes to neurotoxicity and synapse loss by triggering several proinflammatory cascades [7].

We and others have shown that astrocytes and microglia cells express pattern recognition receptors (PRRs), an evolutionarily conserved family of innate immune cell receptors that respond to danger- or pathogen-associated molecular patterns (DAMPs or PAMPs). PRRs bind to different species of Aβ with various affinities [8, 9]. In vitro, Aβ activates microglia by binding to different PRRs, including receptors for advanced glycation end products (RAGE), toll-like receptors (TLRs), scavenger receptors [10–12], and formyl peptide receptors (FPR) [13, 14]. The modulation of glia reactivity, by the interference of PRR signal transduction, might thus be an attractive approach in AD and other neurodegenerative disorders.

Formyl peptide receptors belong to the family of G-protein-coupled receptors (GPCR) that recognize both, exogenous and endogenous “danger” signals, and trigger inflammation and immune responses. The murine FPR gene family has at least six members in contrast to only three in humans. The two most important members, FPR1 and FPR2, are in the focus of the current study. *Fpr1* encodes murine FPR1, which is considered to be the murine orthologue of human FPR, whereas *Fpr2* encodes for receptors that are most similar to human FPR2 (former formyl peptide receptor-like 1 (FPRL1)) [15]. One important characteristic of the FPR family is the broad spectrum of ligands [16]. The first characterized FPR agonist was N-formyl-methionyl-leucyl-phenylalanine (fMLF), isolated from the *E.coli* bacterial cell wall [17]. The results of most of the studies suggest a proinflammatory role of fMLF [18–20]. Further agonists of FPR1 and FPR2 are annexin A1 and its N-terminal peptide Ac2-26, which both predominately exert anti-inflammatory effects [21, 22]. Furthermore, the FPR antagonist N-tert-butyloxycarbonyl-Phe-Leu-Phe-Leu-Phe (Boc2) has been shown to exert anti-inflammatory activities [23, 24]. Of note, the relevance of these FPR modulators as a therapeutic option in AD is currently unknown.

The results of recent studies suggest that FPR2 is involved in Aβ1-42-induced glia cell activation as well as glial Aβ1-42 internalization [25, 26]. FPR2, expressed by astrocytes and microglia, modulates Aβ1-42 uptake and/or signal transduction [13, 14]. In addition, we demonstrated a strong increase of glial FPR1/2 expression in amyloid precursor protein/presenilin 1 (APP/PS1) transgenic mice [27], pointing towards a functional role of the FPR signaling cascade during AD. In this study, we therefore addressed the question whether the modulation of FPR receptors can exert beneficial effect in the APP/PS1 double-transgenic AD model.

## Methods

### Reagents

Ac2-26 and N-tert-butyloxycarbonyl-Phe-Leu-Phe-Leu-Phe (Boc2) were purchased from GenicBio Limited (Shanghai, China). fMLF was obtained from Sigma-Aldrich, Munich, Germany. Ac2-26 and Boc2 were dissolved in 0.9% NaCl solution. fMLF was first dissolved in ethanol and then further diluted in 0.9% NaCl. The different peptides were administered via intraperitoneal injections.

### Mice

Mice were bred and maintained in accordance with local guidelines (LANUV, North Rine-Westphalia). Mice were socially housed in 27 cm × 16.5 cm × 12.5 cm cages (2-5 mice per cage) with enrichment objects and maintained on a standard 12 h cycle of daytime light (6:00-18:00). All interventions were performed during the daytime light cycle. The APP/PS1 double-transgenic mouse model used in this study (APPswe/PS1dE9-Line 85) co-expresses the chimeric mouse/human amyloid precursor protein (APP) 695 harboring the Swedish K670M/N671L mutations (Mo/HuAPPswe), and human presenilin 1 (PS1) with the exon-9 deletion mutation (PS1dE9) under the control of the mouse prion protein promoter [28]. The mouse line was obtained from Jackson Laboratory (B6.Cg-Tg (APPswe, PSEN1dE9) 85Dbo/J; stock number: 005864). Wildtype (WT) littermates on a C57BL/6 background were used as controls. The APP/PS1 mice were generated by mating the single transgenic mice. The WT mice resulting from the mating were used as controls. To limit the number of mice, male and female mice were used in this study. The total number of mice used for behavioral experiments were WT *n* = 26, WT + fMLF *n* = 14, WT + Boc2 *n* = 16, WT + Ac2-26 *n* = 11, APP/PS1 *n* = 21, APP/PS1 + fMLF *n* = 14, APP/PS1 + Boc2 *n* = 15, and APP/PS1 + Ac2-26 *n* = 15. All animal experiments were approved by the Animal Care Committee of the University Hospital of Aachen and by the District Government in Recklinghausen, North Rhine-Westphalia, Germany (reference number 84-02.04.2014.A399).

### Drug treatment

To study protective effects of FPR modulation in the applied AD model, eight-week-old APP/PS1 double-transgenic or WT mice were treated with intraperitoneal injections (i.p.) of either Ac2-26, Boc2, or fMLF twice a week for a period of 20 weeks at the following concentrations: 1 mg/kg body weight for Ac2-26 [29];; 0.5 mg/kg for Boc2; and 40 μg/kg for fMLF [30, 31]. The subsequent assays/quantifications were performed with experimenters blinded to the treatment groups. The mice were sacrificed at a total age of 29 weeks.

### Morris water maze (MWM)

To assess the animals’ long-term memory performance, we used a circular Morris water maze testing paradigm (diameter 120 cm; height 50 cm; and a water temperature of 24 °C). The maze was divided into four quadrants, equipped with four landmarks at the inside of the wall. The transparent escape platform (diameter 10 cm; height 24 cm) was located 1 cm below the water surface. The animals were subjected to the maze each day for 6 consecutive days, with 6 trials per day (3 trials in the morning and 3 trials in the afternoon, respectively). The 3 contiguous trials were performed at 5 min intervals, and the morning and afternoon sessions were separated by a 3-h interval. The experiment was divided into four distinct stages: flagged trials (days 1 and 2, trials 1-12), training trials (days 3 and 4, trials 1-12), test trials (day 5, trials 1-6), and probe trials (day 5, trial 1-6). During the flagged trials, the platform position was clearly indicated by a red flag and all other cues were removed from the maze. The platform was located at 4 variable positions and the animals were placed into the maze at a constant position. The aim of the flagged trials was to inform the mice about the presence of a platform in the water maze. During the training trials, the red flag was removed from the platform and the animals were placed into the maze at different starting positions with a constant platform position. The aim of the training trial was that the mouse memorizes the position of the platform with the help of external visual cues (i.e., different geometric figures). During the test trials, the animals were placed into the maze at a constant platform position (i.e., the last position used during the training trial). During the probe trials, the animals were placed into the maze in the upper left quarter and the platform was removed. The aim of this part of the experiment was to retrieve and check the spatial memory of the mice. In each trial, the mice were placed with the face to the wall, and were allowed to swim freely until they reached the hidden platform. Mice that failed to find the platform within 60 s were subsequently placed onto the platform for 5 s (equal time as successful animals stayed on it). The software package ANY-maze™ software (Stoelting Europe, Dublin, Ireland) was used to track the animals during the MWM procedure and to obtain data regarding their mean distance from the platform, the corrected integrated path length, latency, and path efficiency. Moreover, the software was used to generate track plots for each individual animal [32].

### Immunohistochemistry

For immunohistochemistry, sections were rehydrated and, if necessary, antigens were unmasked with Tris/EDTA buffer (pH 9.0) or citrate (pH 6.0) heating as previously described [33]. The sections were washed in PBS and incubated overnight at 4 °C, with either anti-GFAP (1:75000; RPCA-GFAP, EnCor, Gainesville, FL, USA) or anti-IBA1 (1:10000; CTR6026, Wako, Neuss, Germany) antibodies, diluted in blocking solution (i.e., serum of the species in which the secondary antibody was raised). On the next day, the slides were incubated with 0.3% H_2_O_2_ in PBS for 30 min and then with biotinylated secondary antibodies (1:50; BA-1000; Biozol, Eching, Germany) for 1 h. After a washing step, the slides were incubated with peroxidase-coupled avidin-biotin complex (ABC kit; Vector Laboratories, Peterborough, UK) and subsequently treated with 3,3’-diaminobenzidine (DAKO, Hamburg, Germany) as a peroxidase substrate. Finally, the slides were counterstained with hematoxylin and covered with DePeX (Serva, Heidelberg Germany). For immunofluorescence staining, the slides were incubated with anti-Beta-Amyloid 1-42 (1:150; AB5078P, Merck Millipore, Darmstadt, Germany) or anti-NeuN (1:250; ab17748, Abcam, Cambridge, United Kingdom) antibodies, followed by incubation with anti-rabbit IgG Alexa Fluor 594 secondary antibodies (1:250; A11012, Thermo Fisher Scientific, Dreieich, Germany). To visualize cell nuclei, sections were incubated with Bisbenzimid (1:10.000 in PBS) and then mounted in Immu-Mount (Thermo Fisher Scientific).

### Quantification of immunoreactive cells

Stained and processed sections were digitalized using a Keyence Analysis Software Imaging System (microscope Keyence BZ-9000; Keyence, Neu-Isenburg, Germany). The hippocampus formation and the somatosensory and motor cortices were defined as regions of interest (ROI). In general, three randomly chosen slides were processed and evaluated per stain and experimental animal, respectively.

Different strategies were applied to (semi-) quantify staining intensities. For Aβ1-42 plaques (stained with anti-Beta-Amyloid 1-42 [34]), four different size categories (> 75-125 μm^2^, 125-250 μm^2^, 250-500 μm^2^, > 500 μm^2^) were defined, and the individual area per plaque was quantified using a modified version of the “Analyze particles macro” of ImageJ. Microglial reactivity around the plaques was analyzed in anti-IBA1 stained sections in a circular area around the plaque center (diameter of 50 μm). The chosen diameter of 50 μm was used due to the spatial proximity of Aβ1-42 plaques and therefore the prevention of overlapping. The extent of microglia activation around the plaques is given as “IBA1^+^ area minus the plaque area” in μm^2^, grouped according to their plaque size (categories as mentioned above). In order to quantify neuronal cell densities, the layer V of the motor and somatosensory cortex was delineated in NeuN-stained sections, and NeuN^+^ cells were manually counted using ImageJ. To estimate neuronal cell densities in the dentate gyrus, anti-NeuN fluorescence intensities were determined and expressed as fluorescence intensity in % per hippocampal area. Microglia reactivity was estimated by quantifying cell morphology. To this end, a ramification index (RI) was calculated as published previously by our group [35]. The RI is mathematically defined as: RI = maximum cell projection area (A_p_)/cell area (A_c_). Ramified, resting microglial cells have a large maximum projection area (Ap) and a relatively small cell area (Ac). In contrast, in activated microglial cells or macrophages, the cell area and the maximum projection area are almost identical. Consequently, a fully activated microglia cell takes an RI value close to one [35].

### RNA isolation and realtime RT-PCR

Total RNA was isolated using the peqGold Trifast reagent (30-2010, Peqlab, Erlangen, Germany) according to the manufacturer’s instructions. RNA samples were subsequently reverse-transcribed by a reverse transcriptase kit (#EP0442; Thermo Scientific, Dreieich, Germany) and random hexamer primers (MAN0013111, Thermo Scientific, Dreieich, Germany). The cDNA products were used for SYBR green (Applied Biosystems, Darmstadt, Germany) real-time RT-PCR assays. Gene expression levels were monitored using the StepOne Plus apparatus (Applied Biosystems, Darmstadt, Germany) according to the manufacturer’s protocol. Relative quantification was performed using the ΔΔCt method, which results in ratios between target genes and a housekeeping reference gene index, including TATA box binding protein (*Tbp*), *Rpl13a*, and *m18s*. The primers for glial fibrillary acid protein (*Gfap*) and integrin alpha M (*Itgam*) were manufactured by Qiagen (QT00101145, QT00156471, QuantiTect Primer Assay; Qiagen, Hilden, Germany). The primers for *Tbp*, neprilysin (*Nep*), insulin-degrading enzyme (*Ide*), brain-derived neurotrophic factor (*Bdnf*), nerve growth factor (*Ngf*), glial cell line-derived neurotrophic factor (*Gdnf*), tropomyosin receptor kinase B (*TrkB*), *Rpl13a*, and *m18s* were manufactured by Eurofins MWG Operon (Ebersberg, Germany; for primer sequences see Suppl. Table S1). All reactions were performed with primer-specific pre-evaluated annealing temperatures. The specificity of the amplification reaction was determined by subsequent melting curve analyses. Amplification efficiency was calculated with the LinRegPCR software package (version 12.7).

### Determination of microglial phagocytosis using fluorescence-activated flow cytometry (FAC)

To assess the in vivo Aβ1-42 phagocytosis rate of microglial cells, mice were i.p. injected with 10 mg/kg methoxy-XO4 (Tocris Bioscience, 863918-78-9) in 50% DMSO/50%NaCl (0.9%), pH 12, 3 h before scarification. After transcardial perfusion of the mice with 20 ml PBS, brains were removed, chopped in small pieces and incubated for 1 h in 37 °C in Hanks’ balanced salt solution (HBSS) with 10% FCS and collagenase type IV (0.144 mg/ml, Worthington). By up-and-down pipetting through a 19-G needle, the tissue was mechanically homogenized. After centrifugation (155 g, 4 °C, 10 min without brake, Beckmann Allegra), the pellet was re-suspended in 1 ml 37% Percoll in PBS and gently under layered with 10 ml 70% Percoll in PBS. Centrifugation at 800 g and 4 °C for 25 min without brake leads to a gradient, which contained the microglial cells in the 37/70% Percoll interphase. The cells were carefully removed, diluted with 3 vol PBS and centrifuged at 800 g/4 °C for 25 min. The pellet was then re-suspended in 200 ml PBS. To prevent the binding of the antibodies to the FC-receptors, 1 μl FC-Block (BD Biosciences Cat.: 553142) was added for 10 min on ice. 1 ml HBSS was added, centrifuged at 250 g/4 °C for 5 min and the supernatant was discarded. 50 μl antibody mix (CD11b-APC from Biolegend [101212] 1:50 and CD45-FITC from eBioscience [11-0451-85] 1:50 in HBSS) was added to the pellet and incubated for 30 min on ice. After centrifugation at 250 g at 4 °C for 5 min, the cells were re-suspended in 200 μl PBS. Cell suspensions were analyzed using the cytometer LSRFortessa^TM^ (BD Biosciences, Heidelberg, Germany). For quantification, the CD11b^+^ CD45^+^ population was gated. WT mice injected with methoxy-XO4 were used to determine the methoxy-X04 threshold for non-phagocytosing cells, and unstained WT cells were used to determine background fluorescence intensities [36] (for representative graphs see Suppl. Fig S3).

### Statistical analysis

For statistical calculations, GraphPad Prism 6.0 was used (Graph Pad Software, San Diego, CA, USA). The Kolmogorov-Smirnow test was applied to test for Gaussian distribution of the data. For the non-parametric data, we used the Mann-Whitney or the Kruskal-Wallis test following Dunn’s multiple comparison test. For the normal-distributed data, the significance was calculated with the *t* test or the two-way ANOVA test followed by Turkey post hoc test. The data are presented as the means+/− SEM. The values for realtime RT-PCR analyses are presented as the means of duplicate measurements. A value of *p* < 0.05 was considered as statistically significant.

## Results

### Intraperitoneal administration of Boc2 improves spatial memory in APP/PS1 double-transgenic mice

We have recently shown that the expression of FPR is induced in APP/PS1 double-transgenic mice by both, astrocytes, and microglia cells [27]. To study a possible functional relevance of FPR-activation in AD, we used APP/PS1 double-transgenic and WT mice to test the effect of FPR-modulation via the intraperitoneal injection of different FPR ligands. fMLF (FPR-agonist), Boc2 (FPR-antagonist), and Ac2-26 (anti-inflammatory FPR-modulator) were injected for a period of 20 weeks, and spatial memory was subsequently tested using the Morris water maze paradigm. Gross behavioral inspection of the animals did not reveal any signs of behavioral abnormalities, ruling out negative physical side effects due to the daily i.p. treatment strategy. Furthermore, the physiological condition of the animals was assessed during the initial “flagged trials” with regard to locomotor performance, which did not reveal any difference between the different treatment groups (Suppl. Fig. S1). For the “training trail,” we were not able to find any differences between the groups (Suppl. Fig. S2).

**Fig. 1:**
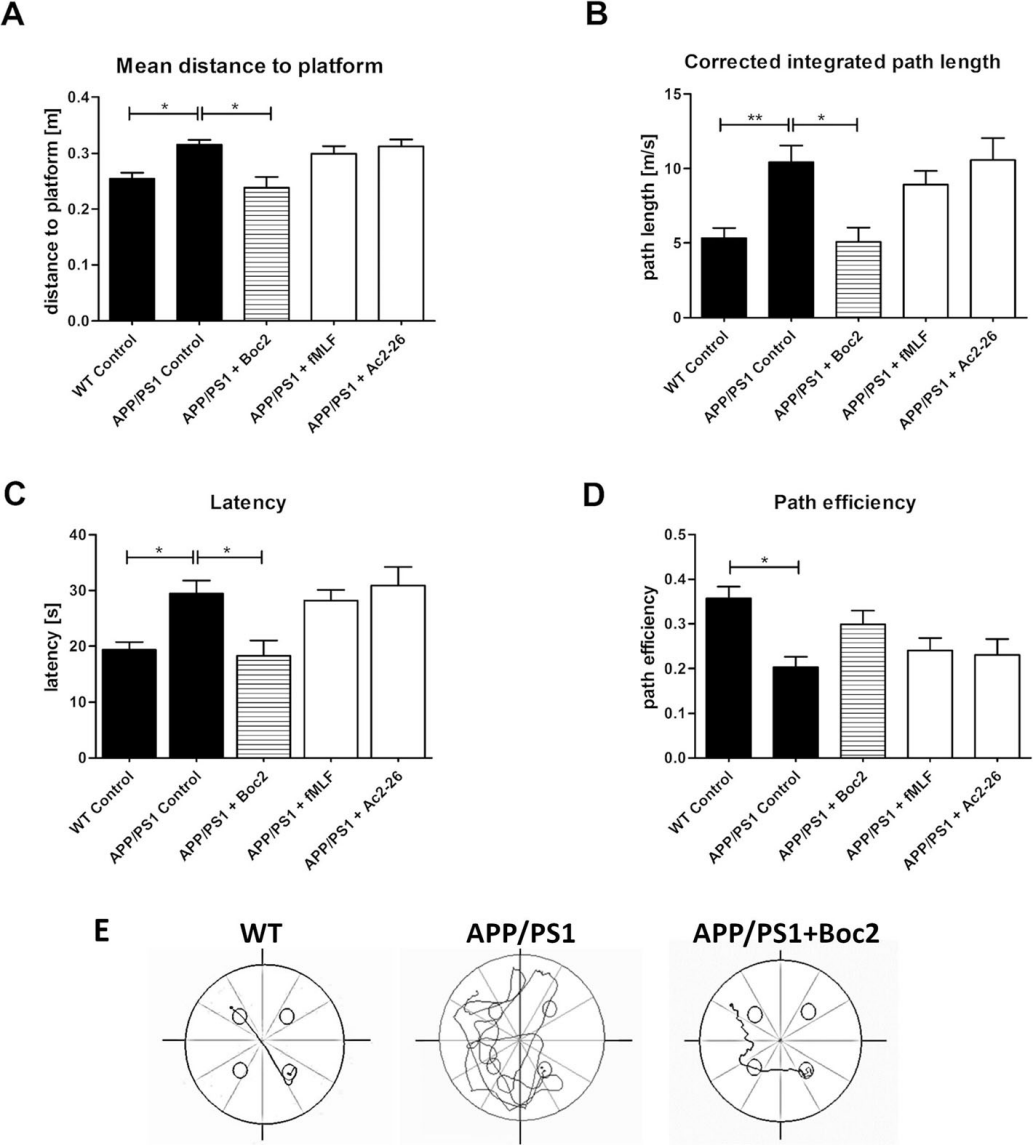
Boc2 treatment of APP/PS1 double-transgenic mice improves spatial memory performance, analyzed by Morris water maze test. The eight-week-old APP/PS1 double-transgenic was injected for a period of 20 weeks with Boc2, fMLF, or Ac2-26. **a** mean distance to platform (m), (**b**) corrected integrated path length (m/s), (**c**) latency (s) time, and (**d**) path efficiency during the test trials were analyzed. (**e**) Representative track plot (test trial) of wildtype or APP/PS1 double-transgenic mice without or with Boc2 treatment. APP/PS1 double-transgenic mice showed a significantly worth performance in long-term memory compared to age-matched WT littermates. Boc2 treatment rescued this effect. The entry of the maze was top left and the platform position (small circle) bottom right. Statistical significances were determined using two-way ANOVAs followed by turkey post hoc test. Data represent mean + SEM; *n* ≥ 14; *p < 0.05, **p < 0.01

To analyze spatial memory performance, the animals were placed into the maze at the same starting position with a constant platform position (i.e., *test trials*), and their migration pathway was automatically analyzed. As demonstrated in Fig. 1, APP/PS1 double-transgenic mice performed significantly worse compared to WT mice. WT mice used a shorter distance to the platform (Fig. 1a; two-way ANOVA with turkey test; *p* < 0.05; WT = 0.25 ± 0.01 vs. APP/PS1 = 0.32 ± 0.01 m), had a shorter integrated path length (Fig. 1b; *p* < 0.01; 5.33 ± 0.67 vs. 10.43 ± 1.12 m/s) and a shorter latency (Fig. 1c; *p* < 0.05; 19.37 ± 1.35 vs. 29.41 ± 2.34 s) which is the time it takes to find the platform. Furthermore, we determined the efficiency in searching the platform which is defined as the actual path length divided by the direct path length. There, WT mice showed a better efficiency in searching the platform compared to APP/PS1 double-transgenic mice (Fig. 1d; *p* < 0.05; 0.36 ± 0.03 vs. 0.2 ± 0.02).

**Fig. 2:**
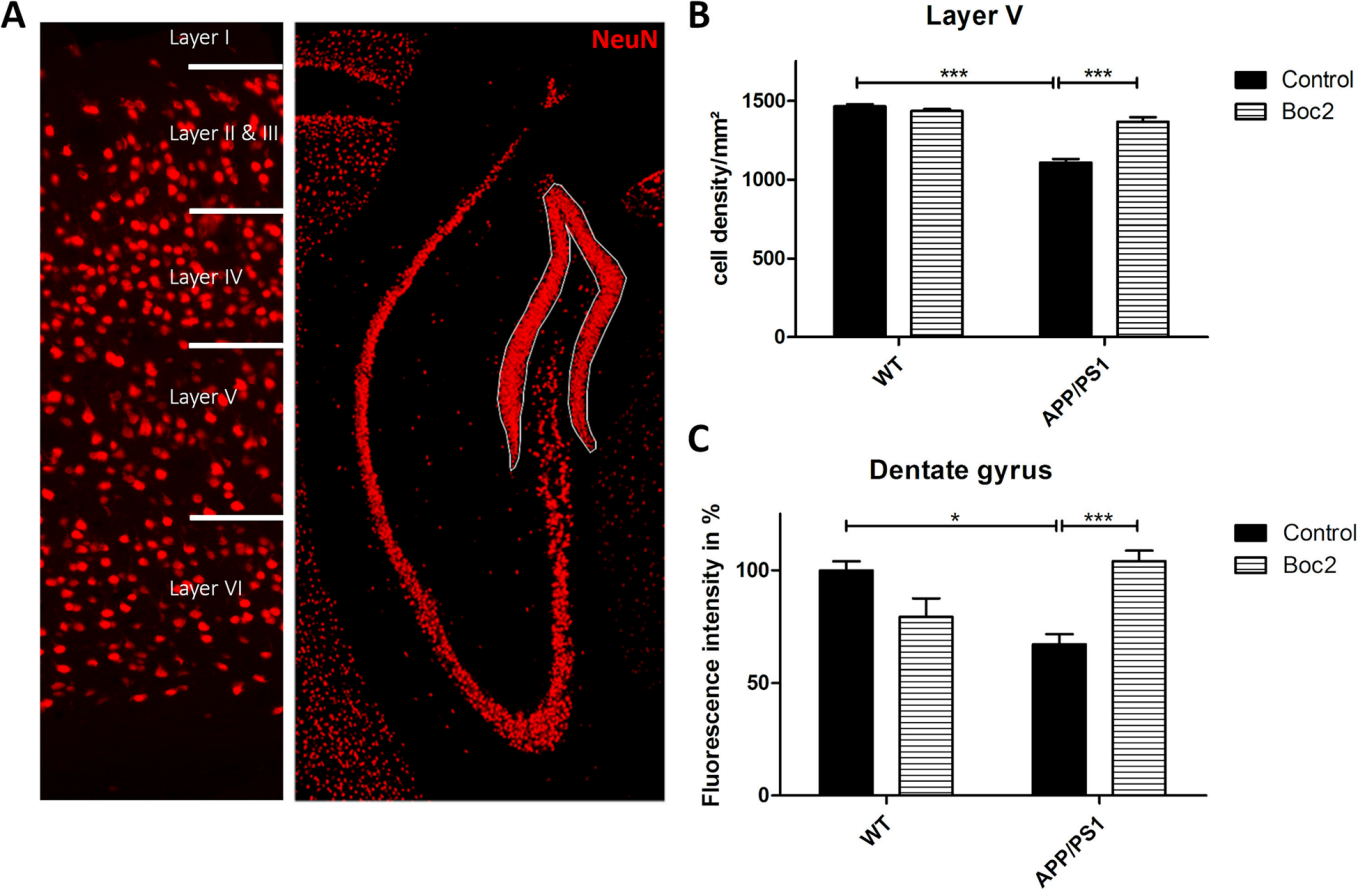
Boc2 treatment ameliorates neuronal loss in APP/PS1 double-transgenic mice. **a** Coronal brain sections of 29-week-old APP/PS1 double-transgenic or WT mice with or without Boc2 treatment were stained with anti-NeuN antibodies to label neuronal cells. Representative images of the cortex (left) and hippocampus (right) are shown. Quantification of NeuN-staining of the (**b**) layer V (cell density/mm^2^) or (**c**) dentate gyrus of the hippocampus (fluorescence intensity %). Statistical significance was determined using Kruskal-Wallis followed by Dunn post hoc test (B/C). Data represent mean + SEM; *n* ≥ 5; *p < 0.05, ***p < 0.001

Next, we tested whether FPR modulation improves the spatial memory performance of our APP/PS1 double-transgenic mice. As demonstrated in Fig. 1, the decline in spatial memory performance was significantly ameliorated in Boc2, but not fMLF or Ac2-26 treated mice. In particular, a significant difference was observed for the parameters “mean distance to platform” (Fig. 1a; *p* < 0.05; 0.24 ± 0.02 m), “corrected integrated path length” (Fig. 2b; *p* < 0.05; 5.07 ± 0.97 m/s), and “latency” (Fig. 1c; *p* < 0.05; 18.27 ± 2.74 s) between APP/PS1 and Boc2-treated APP/PS1 double-transgenic mice. Of note, a comparable protective effect was not observed in APP/PS1 double-transgenic mice treated with either fMLF or Ac2-26. In the next experiments, we therefore focused on the Boc2-treated mice.

### Boc2 is neuroprotective and induces a regenerative brain milieu

Neuronal degeneration is thought to contribute to deficits in spatial learning and memory [37, 38]. To address whether the increased memory performance in Boc2-treated APP/PS1 double-transgenic mice is paralleled by neuronal preservation, we semi-quantified in a next step the density of neuronal cells using NeuN as a marker for mature neurons in the layer V of the cortex and in the dentate gyrus of the hippocampus (Fig. 2a). In line with previous observations [39, 40], mean neuronal densities were significantly decreased in the layer V somatosensory cortex and the dentate gyrus in APP/PS1 double-transgenic mice compared to WT mice (Fig. 2b and c; Kruskal-Wallis followed by Dunn test, *p* < 0.001 and *p* < 0.05; layer V 1465.43 ± 12.99 vs 1107.12 ± 23.45 cells/mm^2^, dentate gyrus 100 ± 7.9 vs. 67.18 ± 4.45 fluorescence intensity). In line with our finding on the behavioral level, neuronal densities were significantly higher in Boc-2 versus vehicle-treated APP/PS1 double-transgenic mice in both investigated brain regions (Fig. 2b and c; *p* < 0.001 and *p* < 0.01; layer V 1107.12 ± 23.45 vs. 1367.85 ± 29.17 cells/mm^2^, dentate gyrus 67.18 ± 4.45 vs. 104.14 ± 4.69 fluorescence intensity).

**Fig. 3:**
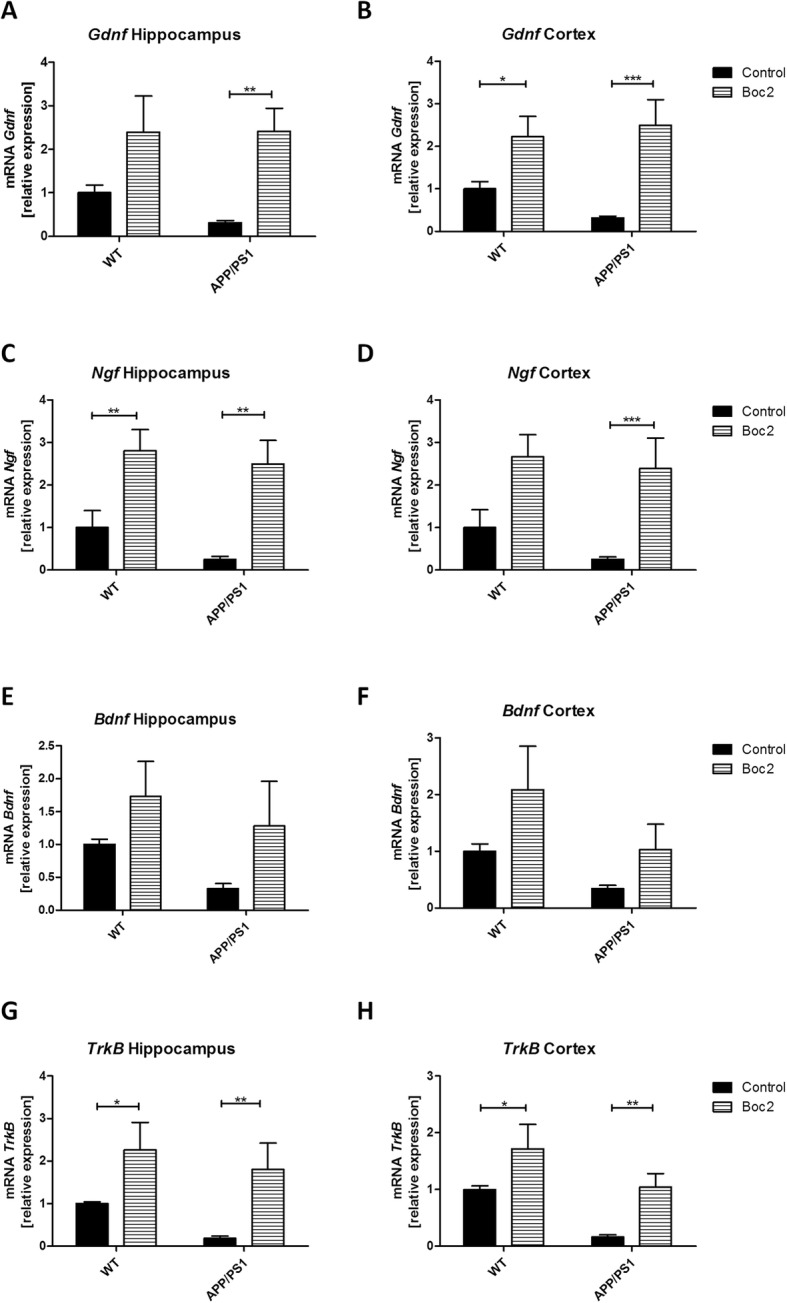
Boc2-treatment induces neurotrophic factor mRNA expression. Analysis of (a, **b**) glial cell line-derived neurotrophic factor (*Gdnf*)**,** (**c, d**) nerve growth factor (*Ngf*), (**e**, **f**) brain-derived neurotrophic factor (*Bdnf*), and (**g**, **h**) tropomyosin receptor kinase B (*TrkB*) mRNA expression levels in the hippocampus and cortex of 29-week-old APP/PS1 double-transgenic and WT mice, with or without Boc2 treatment. Expression levels were analyzed by real-time RT-PCR technology. Statistical significance was determined using two-way ANOVA with turkey test. Data represent mean + SEM; *n* ≥ 5; *p < 0.05, **p < 0.01, ***p < 0.001

Neurotrophic factors play key roles in the development and survival of neurons [41–43]. Therefore, we investigated expression levels of the neurotrophic factors glial cell line-derived neurotrophic factor (*Gdnf*), nerve growth factor (*Ngf*), and brain-derived neurotrophic factor (*Bdnf*) in the different treatment groups. Additionally, tropomyosin receptor kinase B (*TrkB*) expression levels were analyzed, also known as BDNF/NT-3 growth factor receptor. As demonstrated in Fig. 3, expression levels of all investigated mRNA species were found to be lower in APP/PS1 double-transgenic versus WT mice. Boc2-treatment robustly increased the hippocampal and cortical expression of all three growth factors and *TrkB* in both, WT and APP/PS1 double-transgenic mice.

### Boc2 treatment reduces microglial but not astrocyte reactivity in APP/PS1 double-transgenic mice

**Fig. 4:**
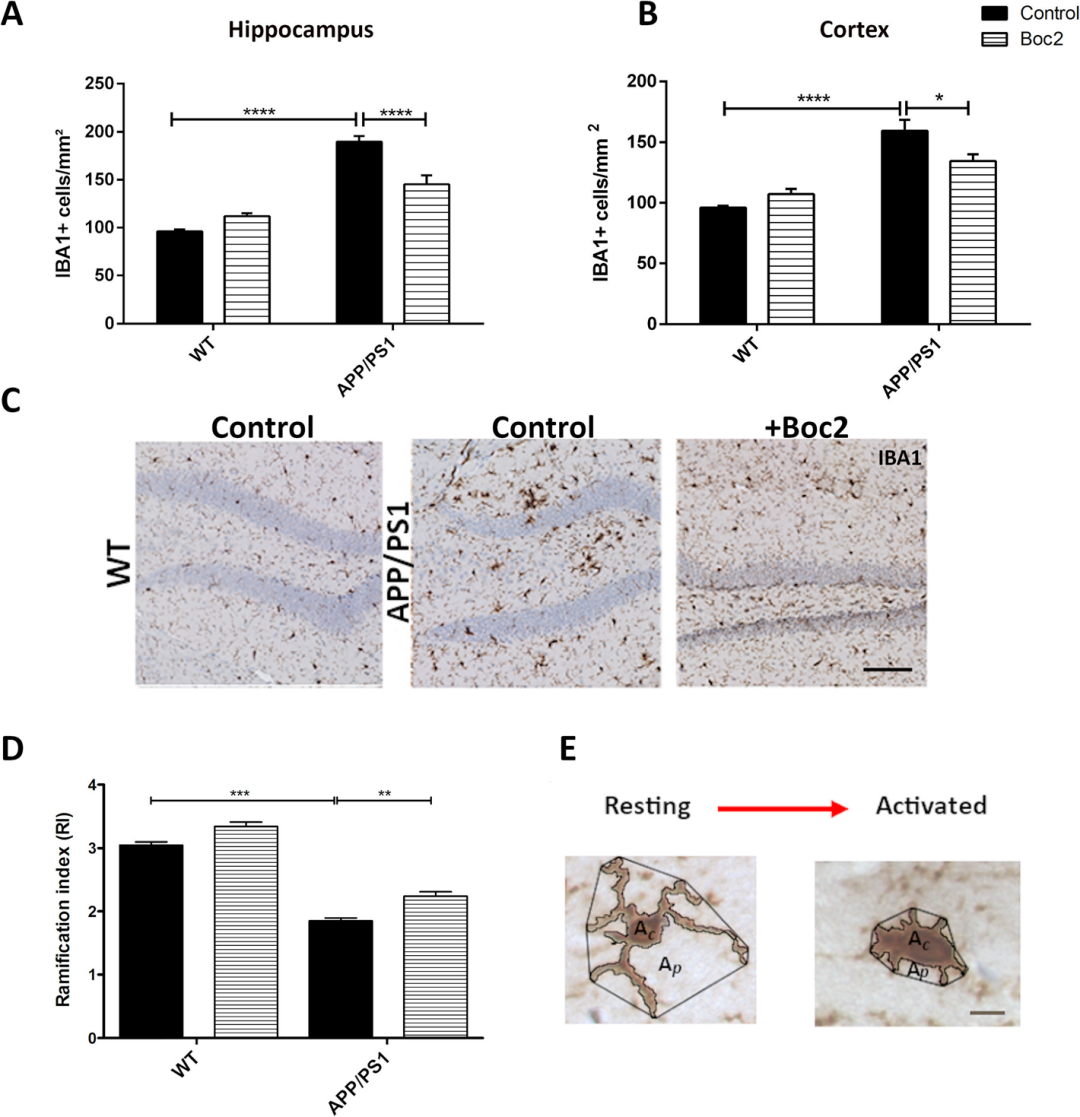
Boc2-treatment ameliorates microglial cell reactivity in APP/PS1 double-transgenic mice. Coronal brain sections of 29-week-old APP/PS1 double-transgenic or WT mice with or without Boc2 treatment were stained with anti-IBA-1 as a marker for microglial cells. Quantification of IBA-1-staining intensities within (**a**) the hippocampus or (**b**) the cortex (cell density/mm^2^). **c** Representative images of the dentate gyrus. **d** Microglia reactivity determined by detailed morphological analysis (i.e., ramification index, RI). Analysis of the RI (Ap/Ac) as a subtle indicator for microglia activation revealed a decrease of RI in APP/PS1 double-transgenic compared to WT mice, which was less severe in Boc2-treated APP/PS1 double-transgenic mice. **e** Analysis of the RI (Ap/Ac) in the hippocampus. Scale bars: (**c**) 50 μm; (**e**) 5 μm. *Ap* projection area *Ac* cell area. Statistical significance was determined using two-way ANOVA with turkey test (A/B) or Kruskal-Wallis followed Dunn test (**d**). Data represent mean + SEM; *n* ≥ 5; *p < 0.05, **p < 0.01, ***p < 0.001

Growth factors are, among other cells, synthetized by astrocytes and can subsequently inhibit microglia reactivity [44]. We, thus, next asked whether the observed increase in growth factor expression levels in Boc2-treated APP/PS1 double-transgenic mice is paralleled by less severe microglia activation. As demonstrated in Fig. 4a/b, a significant increase in microglia cell numbers was observed in the hippocampus and cortex of APP/PS1 double-transgenic versus WT mice (Fig. 4a; two-way ANOVA with turkey test, both *p* < 0.0001; hippocampus WT 96.25 ± 1.89 vs. APP/PS1 189.8 ± 5.93 cells/mm^2^; cortex WT 96.1 ± 1.54 vs. APP/PS1 159.9 ± 9.0 cells/mm^2^). This increase in microglia cell numbers was significantly ameliorated by Boc2 treatment (hippocampus: 145.34 ± 9.3 cells/mm^2^; cortex 134.53 ± 5.49 cells/mm^2^). Another way to analyze microglia activation is to determine their morphology. Resting microglia have thin, ramified processes, and a relatively small cell body. Upon activation, these cells retract their processes and cell swelling occurs. To quantify this aspect, we measured a ramification index (RI), which was calculated by dividing the area around a cell, formed by its processes (Ap), by the internal cell area (Ac; see experimental procedures section). As must be expected, the ramification index of IBA1^+^ cells was significantly decreased in APP/PS1 versus WT mice (Fig. 4d; Kruskal-Wallis followed by Dunn test, *p* < 0.001; WT 3.04 ± 0.05 vs. APP/PS1 1.85 ± 0.05 RI). Of note, the ramification index was significantly higher in Boc2-treated compared to vehicle-treated APP/PS1 double-transgenic mice (*p* < 0.001; 1.85 ± 0.05 vs. 2.24 ± 0.07).

Furthermore, astrocyte reactivity was analyzed in anti-GFAP stained sections. Comparable to what we observed for microglia cells, a significant increase in astrocyte cell numbers was observed in the hippocampus and cortex of APP/PS1 double-transgenic versus WT mice (Suppl. Fig. S4A; two-way ANOVA with turkey test, *p* < 0.0001; hippocampus WT 105.7 ± 2.29 vs. APP/PS1 140.35 ± 3.36 cells/mm^2^; cortex WT 5.58 ± 1.0 vs. APP/PS1 68.37 ± 8.37 cells/mm^2^). In contrast to what we observed for microglia cells, astrocyte reactivity in APP/PS1 double-transgenic mice was not ameliorated by Boc2 treatment. Furthermore, *Gfap* expression levels were found to be increased in APP/PS1 double-transgenic versus WT mice, with no difference in Boc2-treated mice. In summary, Boc2 treatment modulated microglia but not astrocyte reactivity.

### FPR modulation fine-tunes microglia function in AD mice

**Fig. 5:**
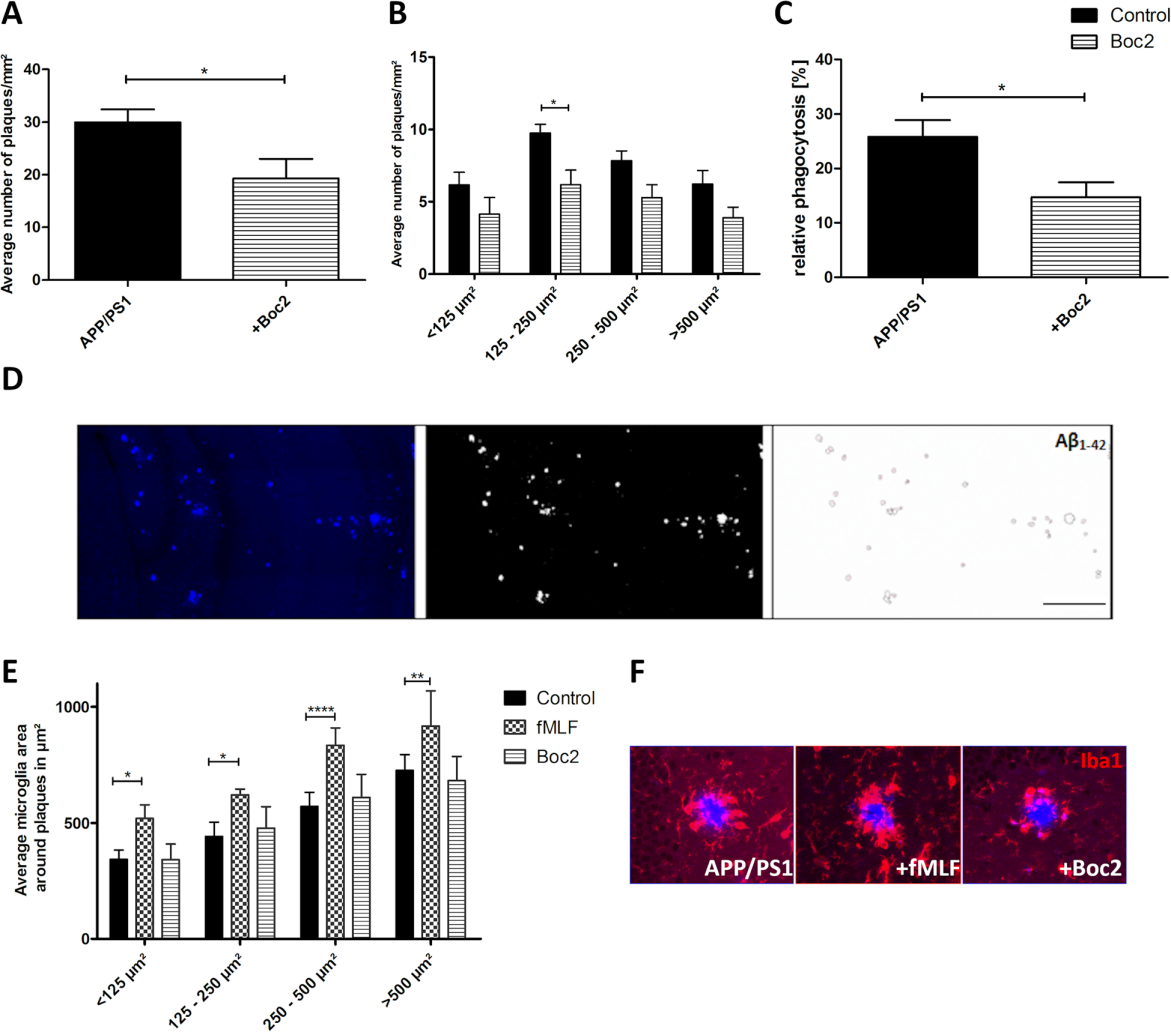
Boc2-treatment reduces plaque load and phagocytosis rate of Aβ in APP/PS1 double-transgenic mice. Coronal brain sections of 29-week-old APP/PS1 double-transgenic mice with or without Boc2 treatment stained with anti-Beta-Amyloid 1-42 were used for the determination of plaque load. **a** Analysis of the total hippocampal Aβ plaque load (number/mm^2^). **b** The average number of plaques/mm^2^ grouped in four different size categories (< 125 μm^2^, 125-250 μm^2^, 250-500 μm^2^, and > 500 μm^2^). Plaque numbers were analyzed in anti-Beta-Amyloid 1-42 processed sections. **c** Aβ phagocytosis rate (in %), analyzed by flow cytometry of microglia isolated from APP/PS1 double-transgenic mice with or without Boc2 treatment 3 h after intraperitoneal injection of methoxy-XO4. **d** Representative images of hippocampal plaques analysis demonstrating the evaluation process with ImageJ. Scale bar: 250 μm. **e** Average microglial cell area (μm^2^) around the Aβ plaques, visualized by anti-IBA-1 stains in the hippocampus of APP/PS1 double-transgenic mice with or without Boc2 or fMLF treatment. **f** Representative image of a plaque stained with anti-IBA-1 (red) and anti-beta-Amyloid 1-42 (blue). Statistical significances were determined using Mann-Whitney *U* test (**a**), Kruskal-Wallis followed Dunn test (**b**), two-way ANOVA with turkey test (**d**), or t-test (**f**). Data represent mean + SEM; *n* ≥ 7; *p < 0.05, **p < 0.01, ***p < 0.001 as indicated

Since we observed microglia but not astrocyte modulation by Boc2 treatment, we focused in subsequent mechanistic studies on the interplay of Aβ1-42 with microglia cells. The typical hallmark of AD is the aggregation of Aβ1-42 peptides to solid plaques. Therefore, in a next step, we analyzed the hippocampus plaque load. As demonstrated in Fig. 5a, the total hippocampal plaque load was significantly reduced in Boc2- compared to vehicle-treated APP/S1 double-transgenic mice (Fig. 5a; Mann-Whitney *U* test, *p* = 0.027; 29.98 ± 2.47 vs. 19.32 ± 3.68 plaques/mm^2^). To learn which plaque types (small versus large) are preferentially reduced by Boc2 treatment, we quantified the numbers of small (> 75-125 μm^2^), medium (125 250 μm^2^), large (250 500 μm^2^), and very-large (> 500 μm^2^) sized plaques. As shown in Fig. 5b, all plaque sizes showed reduced numbers in Boc2-treated APP/PS1 double-transgenic mice, which was significant for medium-sized plaques (Kruskal-Wallis followed Dunn test, *p* < 0.05; 9.75 ± 0.6 vs. 6.172 ± 1.0 plaques/mm^2^). Next, we analyzed microglia reactivity in the close vicinity of the Aβ1-42 plaques. First, as an internal reference, we analyzed peri-plaque microglia reactivity in fMLF-treated mice, which was shown to be not protective in our AD model (compare Fig. 1). As demonstrated in Fig. 5e, we found severe microgliosis in close vicinity to the plaques in fMLF-treated mice. The peri-plaque microgliosis was less intense in Boc2-treated APP/PS1 double-transgenic mice compared to fMLF-treated mice (Fig. 5e and f).

**Fig. 6:**
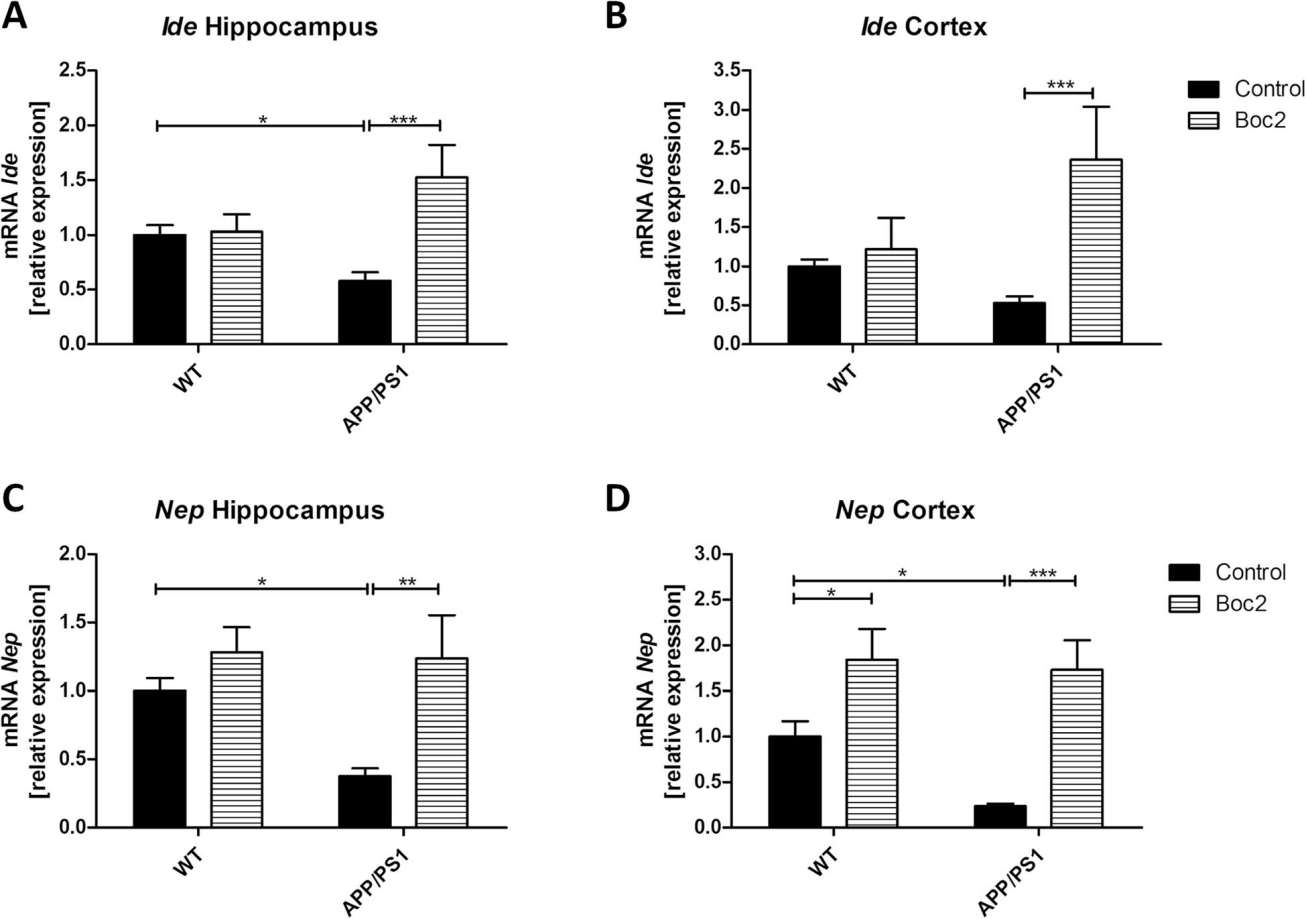
Boc2-treatment increases the expression of Aβ-degrading enzymes in APP/PS1 double-transgenic mice. (**a**, **b**) insulin-degrading enzyme (*Ide*) and (c, d) neprilysin (*Nep*) mRNA expression in the hippocampus and cortex of 29-week-old APP/PS1 double-transgenic mice or WT mice with or without Boc2 treatment. Statistical significance was determined using two-way ANOVA with turkey test. Data represent mean + SEM; *n* ≥ 6; *p < 0.05, **p < 0.01, ***p < 0.001 as indicated

To analyze whether Aβ1-42 degradation or phagocytosis is responsible for the observed reduction in Aβ1-42 hippocampal plaque load, we measured, on the one hand, the phagocytic activity of microglia cells in Boc2-treated APP/P1 double-transgenic mice, and on the other hand, we determined the expression levels of the Aβ-degrading enzymes insulin-degrading enzyme (*Ide*) and neprilysin (*Nep*) in isolated brain tissues. As demonstrated in Fig. 5c, Boc2 significantly reduced the phagocytic activity of Aβ1-42 (Fig. 5c; *t* test, *p* < 0.05; APP/PS1 25.84 ± 3.04 vs. APP/PS1 + Boc2 14.68 ± 2.77 %). Furthermore, expression levels of both degrading enzymes were reduced in APP/PS1 compared to WT mice (Fig. 6; two-way ANOVA with turkey test; *p* < 0.001, *Ide* hippocampus 0.58 ± 0.08 vs. 1.52 ± 0.3; *p* < 0.001, *Ide* cortex 0.53 ± 0.08 vs. 2.36 ± 0.7; *p* < 0.01, *Nep* hippocampus 0.38 ± 0.06 vs. 1.24 ± 0.32; *p* < 0.001, *Nep* cortex 0.23 ± 0.03 vs. 1.73 ± 0.32).

## Discussion

Here, we demonstrate a broad spectrum of beneficial Boc2 effects in a model of AD including improved cognition, decreased microglial cell activation, increased neurotrophic factor expression, preservation of neuronal loss, and increased expression of Aβ-degrading enzymes together with a reduced amyloid plaque load. These findings support the notion that the FPR modulator Boc2 may be a powerful therapeutic option in AD.

This study is a continuation of our previous works were we demonstrated that FPRs are involved in prion protein and Aβ1-42-mediated signaling cascades [13, 14, 45]. We and others have previously shown that the expression of both, *Fpr1* and *Fpr2*, is altered in various inflammatory and neurodegenerative animal models including APP/PS1 double-transgenic [27], in a model of acute, metabolic oligodendrocyte injury [33], or in a model of bacterial meningitis [46]. In general, FPRs are not only involved in host defense against pathogens but also in sensing internal harmful molecules such as mitochondrial peptides, f-MLKLIV, serum amyloid A, or prion protein [45, 47]. One important characteristic of FPRs is that they can be activated by both, pro- and anti-inflammatory ligands. For example, the bacterial cell wall component fMLF, which is classically regarded as a pro-inflammatory mediator, binds FPR and induces downstream signaling cascades. On the other hand, Ac2-26, which is a bioactive N-terminal fragment of the endogenous protein annexin A1 [47], harbors several anti-inflammatory or protective properties [30, 48, 49]. Here, we were now interested, whether or not FPR modulation exerts beneficial effects in a pre-clinical AD model.

In this study, APP/PS1 double-transgenic mice were treated for 20 weeks with the FPR antagonist Boc2, the pro-inflammatory bacterial cell wall component fMLF or the anti-inflammatory FPR agonist Ac2-26 to analyze the effects of an FPR modulation in AD. The FPR antagonist Boc2, but not the FPR agonists fMLF or Ac2-26 exerted beneficial effects in the applied AD model. While it has been shown (i) that different formyl peptides, such as fMLF, NfMA, or NfM can induce the release of the pro-inflammatory, inflammasome-associated cytokine IL-1β from macrophages [50], (ii) that Aβ1-42 can activate macrophages in a FPR2-dependent manner, and (iii) that FPRL1 is expressed at high levels by inflammatory cells infiltrating senile plaques [25], to the best of our knowledge no data are available regarding the effect of *Fpr* deletion in AD mice. Our results indicate that FPR activation by amyloid or other DAMPs activates destructive events in AD, and that the inhibition of the FPR receptor ameliorates such destructive processes. These observations are in line with findings of a recent report where *Fpr2*-deficient mice were protected against streptozotocin-induced cognitive deficits [51], which has been suggested to recapitulate distinct aspects of the AD pathology.

To our great surprise, Ac2-26-treatment did not show any effects in our applied AD animal model. Ac2-26 decreased ocular inflammation in vitro and in vivo [29] showed cardioprotective actions against myocardial infarction [48] or improved skin heterologous transplantation [52]. With respect to the CNS, Ac2-26 showed anti-inflammatory and neuroprotective effects in a model of pilocarpine-induced status epilepticus [49] or resolved cerebral inflammation in sepsis [30]. Increased susceptibility of annexin-A1 null mice to nociceptive pain is indicative of a spinal antinociceptive action of annexin-A1 [53]. One possibility why Ac2-26 failed to demonstrate protective effects in our study is that it was not able to penetrate into the brain parenchyma. Imaging studies in patients with early AD demonstrated blood-brain-barrier breakdown in several gray and white matter regions [54–56]. Comparably, vascular pathology was observed in APP/PS1 double-transgenic mice [57, 58]. In a model of bacterial meningitis, induced by *Streptococcus pneumoniae* infection, treatment with Ac2-26 clearly showed protective effects (manuscript in preparation). In contrast to APP/PS1 double-transgenic mice, the blood-brain barrier is severely impaired in meningitis [59] allowing Ac2-26 to exert beneficial effects in the brain. In general, the mode of action of Ac2-26 on the level of the receptor is equally poorly understood. One study showed Ac2–26-induced internalization of FPR in vitro [60]. In addition, Ac2-26 suppresses TNFα-induced inflammatory responses via inhibition of Rac1-dependent NADPH oxidase [61] or triggers ERKs phosphorylation via FPR [47]. Further studies are required to test brain penetrance of Ac2-26 in the applied animal model, and to analyze its effect on glia cells and neurons on the receptor level.

Although the failure of Ac2-26 to ameliorate the pathology in APP/PS1 double-transgenic mice was somewhat unexpected, Boc2, which is an antagonist of the FPR [29], clearly ameliorated the disease course in APP/PS1 double-transgenic mice. Twenty-eight weeks old APP/PS1 double-transgenic mice showed cognitive and memory impairments in a MWM test, which was ameliorated by Boc2 treatment. The MWM test is an accepted paradigm to investigate the severeness of cognitive dysfunction in rodents. Navigating safely through the environment is vital to the survival of humans and animals. The ability to do this depends on appropriate cognitive performances, in particular learning and remembering locations. This capacity is encoded in the CNS by two distinct systems, allocentric navigation and egocentric navigation. Different brain structures are involved in allocentric navigation including the hippocampus, the entorhinal cortex, and neighboring structures. This form of memory can be assessed in laboratory animals in many ways, but the most frequently applied method is the Morris water maze [32]. Our finding that Boc2-treatment improves the performance of APP/PS1 double-transgenic mice in the MWM might, thus, have direct relevance for AD patients.

The cognitive loss in the course of AD is paralleled by a reduction of intracerebral growth factor levels such as BDNF [62]. The deficiency of BDNF has recently identified to be key for motor-learning by promoting learning-related synapse formation [63]. Furthermore, preclinical studies have demonstrated the beneficial effects of several neurotrophic factor small-molecule mimetics, particularly BDNF and ciliary neurotrophic factor mimetics [64]. As demonstrated in Fig. 3 in this manuscript, the expression of several neurotrophic factors, among *Bdnf*, was found to be increased in Boc2-treated mice. Of note, such a growth factor stimulating effect was observed in both, WT and AD mice. This means that even under physiological conditions modulation of FPR-signaling cascades, especially by Boc2-treatment, might exert beneficial effects. Importantly, the induction of growth factor expression was not observed in Ac2-26 nor fMLF-treated mice (data not shown), indicating that this positive effect is Boc2 specific. In line with the observation of a preservation of cognitive function in Boc2-treated mice and expression induction of neurotrophic factors, Boc2 treatment ameliorated neuronal loss in cortical and subcortical structures. Although not formally demonstrated in this study, the observed induction of growth factor expression in even WT mice strongly suggests that at least Boc2 can cross the intact blood-brain-barrier. Whether the same is true for AC2-26 and fMLF remains to be clarified in future studies.

Glia cells, in particular microglia and astrocytes, are considered to represent the local innate immune system of the CNS [7, 65, 66]. The activation of microglia represents a common pathological feature of several neurodegenerative diseases, including AD. Microglia-derived proinflammatory cytokines can cause neuroinflammation and neurotoxicity in the brain [7]. Our results show that Boc2 inhibits microgliosis in APP/PS1 double-transgenic mice. This effect was robust and could be observed in the cortex and the hippocampus. Amelioration of microgliosis was evident on both the morphological and expression level. Whereas activated microglial cells in vehicle-treated mice showed an amoeboid phenotype, microglia in Boc2-treated APP/PS1 double-transgenic mice demonstrated a more resting phenotype with ramified processes and relatively small cell bodies. Astrocytes, the second innate immune cell type of the brain, can equally respond to pathological stimuli through reactive gliosis. Comparable to myeloid cells, astrocytes surround Aβ plaques, and studies using transgenic mice exhibiting cerebral amyloidosis have shown that astrocyte activation occurs early in the course of the disease [2]. In contrast to our observations for microglia, the extent of astrocyte activation in APP/S1 mice was not reduced by Boc2-treatment. Whether or not astrocyte-derived BDNF release induces microglia silencing in this model remains to be clarified in future studies [44].

Aß1-42 is formed from the breakdown of amyloid precursor protein and is thought to be especially toxic to neurons [2, 67]. As demonstrated in Fig. 5, Boc2 treatment induced a significant reduction of the entire plaque load in APP/PS1 mice. A more sophisticated analysis of the different plaque sizes revealed that especially medium-sized plaques are reduced in numbers by Boc2 treatment. Aβ acts as danger-associated molecular patterns (DAMP) which can bind to different DAMP-receptors, among FPR, which are expressed by microglia and astrocytes [13, 14, 25, 27]. Aß binding to its receptors induced the expression of various pro-inflammatory mediators [68]. In addition, Aβ has been shown to be cleared by microglia in vitro and in vivo through receptor-mediated phagocytosis and degradation [69, 70]. Here we verify, by flow cytometry analyses, the finding that Aß is phagocytosed by microglia in vivo, and show that Boc2 treatment reduces, and not increases, Aβ phagocytosis of microglial cells in APP/PS1 double-transgenic mice. This means that the reduced Aβ-load in Boc2-treated mice is not due to an induction of Aβ phagocytosis. These results could be explained due to the specificity of Boc2. It displays inhibitory effects on FPR1 > FPR2 [16]. It has been suggested that Aβ-phagocytosis is mediated through FPR2 rather than FPR1 [26], which could explain why Boc2 does not modify Aβ-phagocytosis rates. Another underlying mechanism for the observed amyloid plaque load is that Boc2 induces the expression of Aβ-degrading enzymes. As demonstrated in Fig. 6, the expression of the Aβ-degrading enzymes *Nep* and *Ide* was decreased in APP/PS1 double-transgenic compared to WT mice. These findings are in line with another study showing that microglia from transgenic AD mice had reductions in the levels of Aβ-degrading enzyme [71]. Most importantly, the reduced expression of both Aβ-degrading enzymes was not observed in Boc2-treated APP/PS1 double-transgenic mice, indicating that Boc2 could stabilize the Aβ degradation machinery, resulting in reduced amyloid levels in the CNS. However, by immunohistochemistry using anti-IDE antibodies, we were not able to demonstrate higher numbers of IDE-expressing cells (data not shown). Clearly, more studies are needed to clarify how Boc2-treatment reduces the amyloid plaque load in APP/PS1 mice.

## Conclusion

If AD mice are treated at an early disease stage with Boc2, amyloid load is reduced, the cognition is preserved and neurodegeneration is ameliorated. We suggest that on the one hand, Aß binds to FPR and other DAMP receptors and induces the expression of neurotoxic molecules, finally leading to neuronal cell loss and cognitive decline. Boc2 blocks Aß binding and inhibits such deleterious consequences. Furthermore, Boc2 induced the expression of Aβ-degrading enzymes potentiating its protective effects.

## Supplementary information

**Additional file 1: Fig. S1.** Physical condition of animals is not affected neither in APP/PS1 mice nor by FPR ligands. To analyze the animals physical condition we used the Morris water maze. The average speed [m/s] of the flagged trials were recorded for each animal. There were no differences regarding the condition between the groups (two-way ANOVA with Turkey’s post hoc test). The data represent mean + SEM, n>11. **Fig. S2.** Illustration of Morris water maze training trials To investigate long-term memory we used the Morris water maze test. To analyze the learning process we used the latency time [s] of the training trials (1-12). Learning curves are separated for a better overview in A) WT group and B) in APP/PS1 mice group. The findings demonstrated that the animals improved their performance over one day (trial 1-6). Data represent mean + SEM; n > 11. **Fig. S3.** Quantification of amyloid-β 1-42 phagocytosis of microglial cells by flow cytometry. Cells were isolated from adult mice brains 3h after intraperitoneal injection of methoxy-04. Exemplary graphs of each group shows the evaluation of the phagocytosis rate (Q2). **Fig. S4.** FPR modulation does not affect astrocytes in APP/PS1 mice A) GFAP positive cells/mm² in the hippocampus where increased from WT control to APP/PS1 control mice.B) Also in the cortex we could see the same increased amount of GFAP positive cells in APP/PS1 control mice compared to WT control (n>15) C) Exemplary anti-GFAP staining’s of WT, APP/PS1 and APP/PS1+Boc2 mice in the cortex. D) Relative expression of Gfap mRNA in the hippocampus showed no differences but E) in the cortex we detected an increased Gfap mRNA expression in APP/PS1 control mice (n>6, ). Scale bar c 50 μm. Shown are the mean values of each group with SEM. Two-way ANOVA with turkey test *p <0.05 **p<0.01 ***p<0.001 ****p<0.0001. **Table S1.** Used primer pairs with sequences, specific annealing temperature and supplier information.

## Data Availability

The datasets used and/or analyzed during the current study are available from the corresponding author on reasonable request.
