# Supplementary material for: Inhibition of formyl peptide receptors improves the outcome in a mouse model of Alzheimer disease
Source: J Neuroinflammation. 2020 Apr 24;17:131. doi: 10.1186/s12974-020-01816-2 (PMC7181500; doi:10.1186/s12974-020-01816-2)
Supplement: Supplementary file 1 — Additional file 1: Fig. S1. Physical condition of animals is not affected neither in APP/PS1 mice nor by FPR ligands. To analyze the animals physical condition we used the Morris water maze. The average speed [m/s] of the flagged trials were recorded for each animal. There were no differences regarding the condition between the groups (two-way ANOVA with Turkey’s post hoc test). The data represent mean + SEM, n>11. Fig. S2. Illustration of Morris water maze training trials To investigate long-term memory we used the Morris water maze test. To analyze the learning process we used the latency time [s] of the training trials (1-12). Learning curves are separated for a better overview in A) WT group and B) in APP/PS1 mice group. The findings demonstrated that the animals improved their performance over one day (trial 1-6). Data represent mean + SEM; n > 11. Fig. S3. Quantification of amyloid-β 1-42 phagocytosis of microglial cells by flow cytometry. Cells were isolated from adult mice brains 3h after intraperitoneal injection of methoxy-04. Exemplary graphs of each group shows the evaluation of the phagocytosis rate (Q2). Fig. S4. FPR modulation does not affect astrocytes in APP/PS1 mice A) GFAP positive cells/mm² in the hippocampus where increased from WT control to APP/PS1 control mice.B) Also in the cortex we could see the same increased amount of GFAP positive cells in APP/PS1 control mice compared to WT control (n>15) C) Exemplary anti-GFAP staining’s of WT, APP/PS1 and APP/PS1+Boc2 mice in the cortex. D) Relative expression of Gfap mRNA in the hippocampus showed no differences but E) in the cortex we detected an increased Gfap mRNA expression in APP/PS1 control mice (n>6, ). Scale bar c 50 μm. Shown are the mean values of each group with SEM. Two-way ANOVA with turkey test *p <0.05 **p<0.01 ***p<0.001 ****p<0.0001. Table S1. Used primer pairs with sequences, specific annealing temperature and supplier information. [file 12974_2020_1816_MOESM1_ESM.docx]

Supplementary Information for

**Inhibition of formyl peptide receptors improved outcome in a mouse model of Alzheimer disease**

Nicole Schröder, Anja Schaffrath, Josua A. Welter, Tim Putzka, Angelika Griep, Patrick Ziegler, Elisa Brandt, Sebastian Samer, Michael T. Heneka, Hannes Kaddatz, Jiangshan Zhan, Eugenia Kipp, Thomas Pufe, Simone C. Tauber, Markus Kipp and Lars-Ove Brandenburg

Prof Dr. Lars-Ove Brandenburg

Email: lbrandenburg@ukaachen.de

**This PDF file includes:**

Figs. S1 to S4

Table S1

**
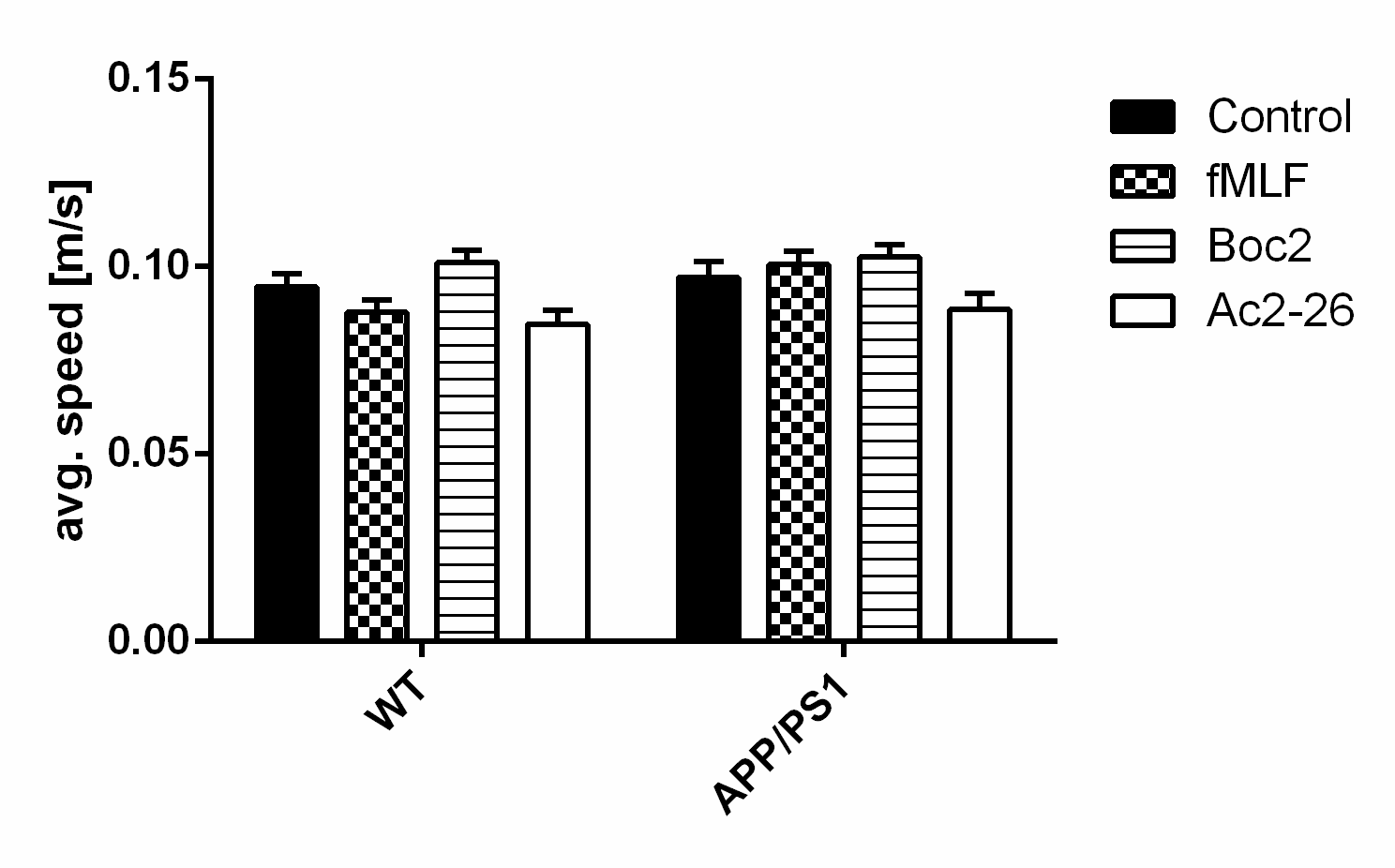
**

**Fig. S1.** **Physical condition of animals is not affected neither in APP/PS1 mice nor by FPR ligands.** To analyze the animals physical condition we used the Morris water maze. The average speed [m/s] of the flagged trials were recorded for each animal. There were no differences regarding the condition between the groups (two-way ANOVA with Turkey’s post hoc test). The data represent mean + SEM, n>11.

**A**

**B**


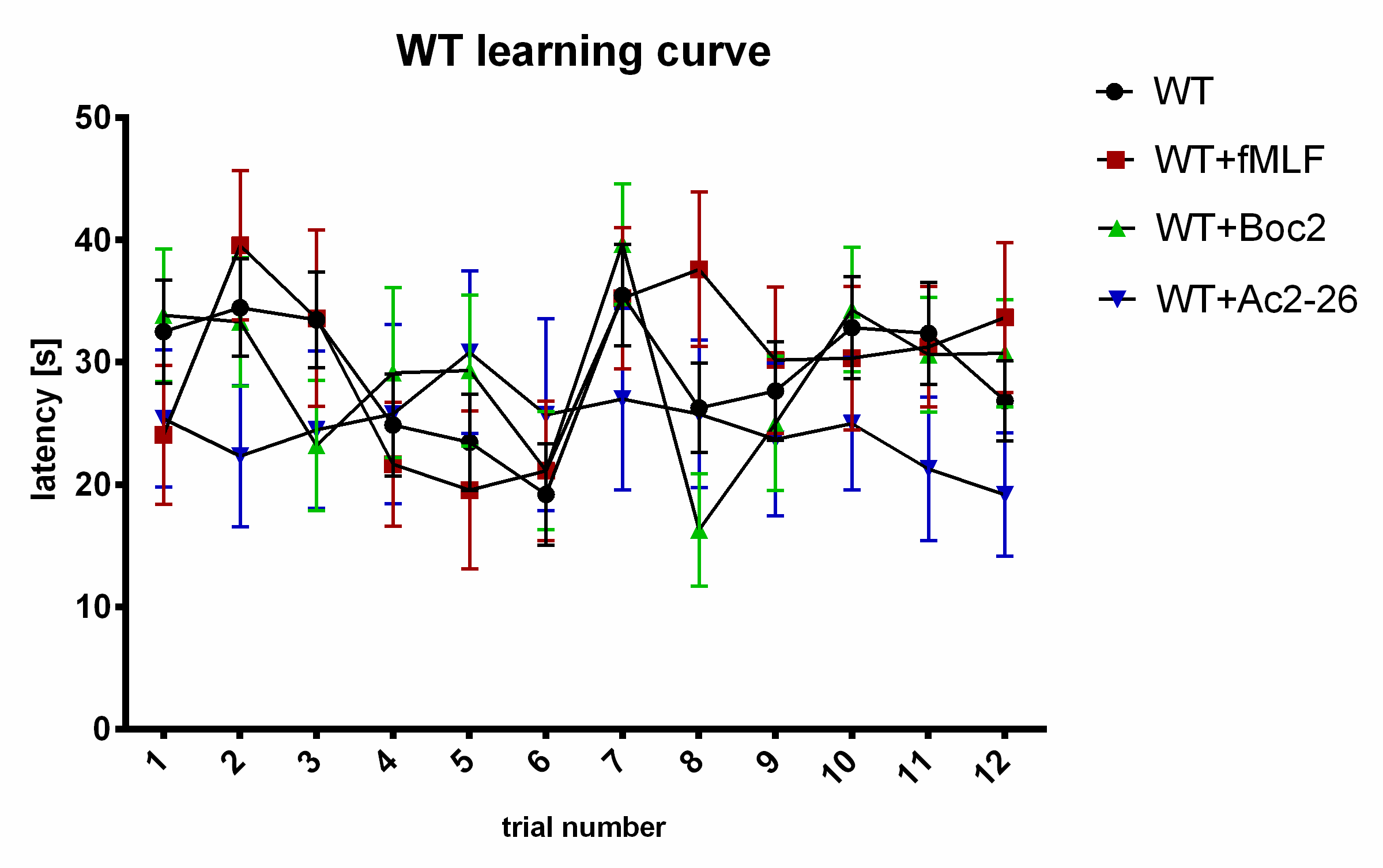


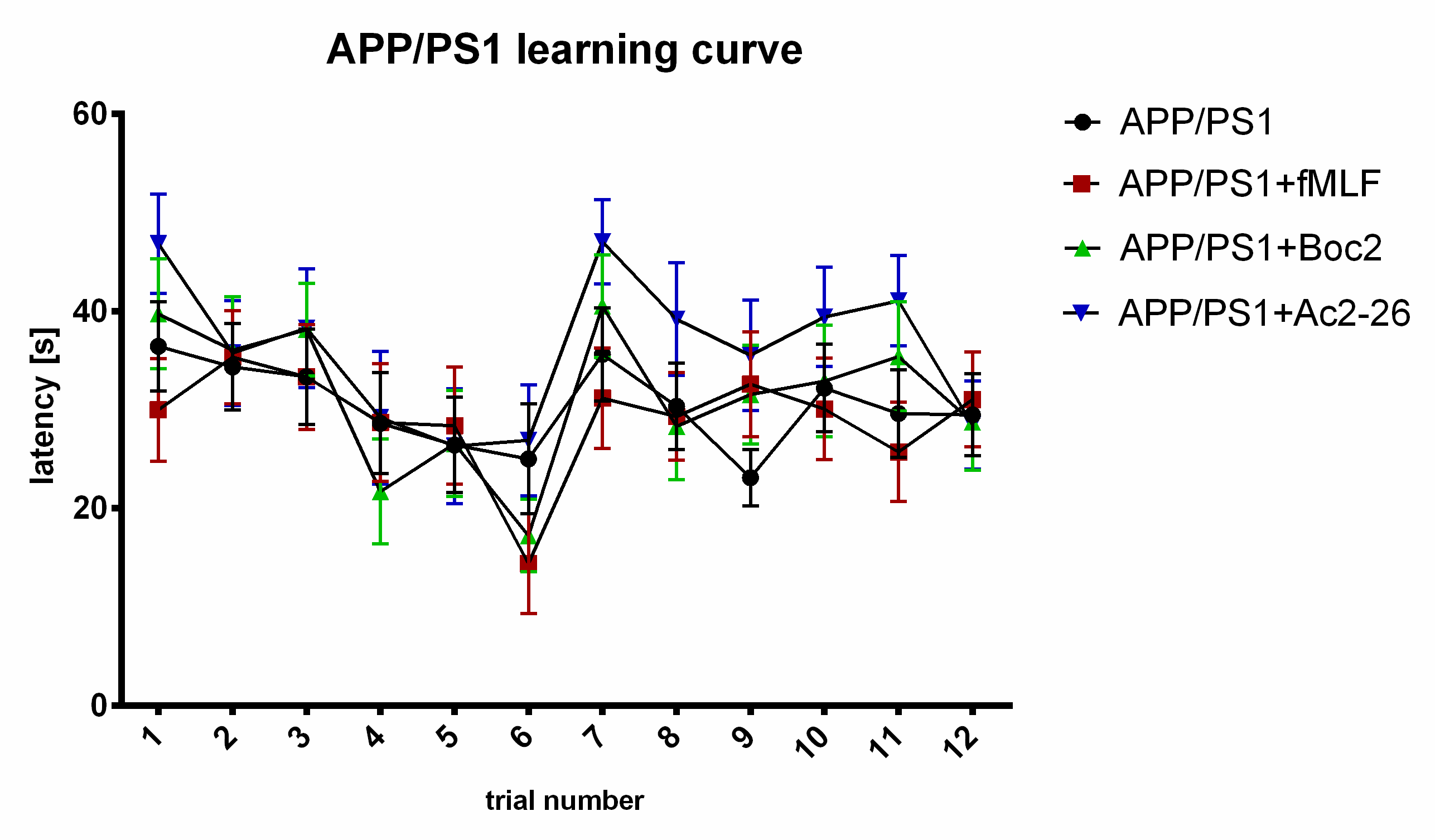


**Fig. S2.** **Illustration of Morris water maze training trials** To investigate long-term memory we used the Morris water maze test. To analyze the learning process we used the latency time [s] of the training trials (1-12). Learning curves are separated for a better overview in A) WT group and B) in APP/PS1 mice group. The findings demonstrated that the animals improved their performance over one day (trial 1-6). Data represent mean + SEM; n > 11.


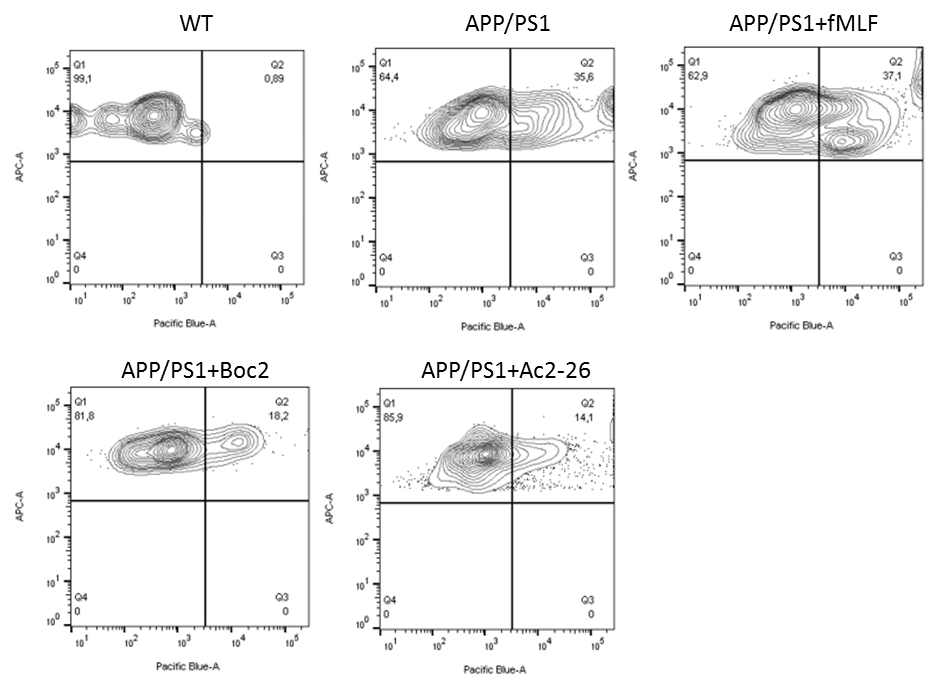


**Fig. S3. Quantification of amyloid-β 1-42 phagocytosis of microglial cells by flow cytometry.** Cells were isolated from adult mice brains 3h after intraperitoneal injection of methoxy-04. Exemplary graphs of each group shows the evaluation of the phagocytosis rate (Q2).


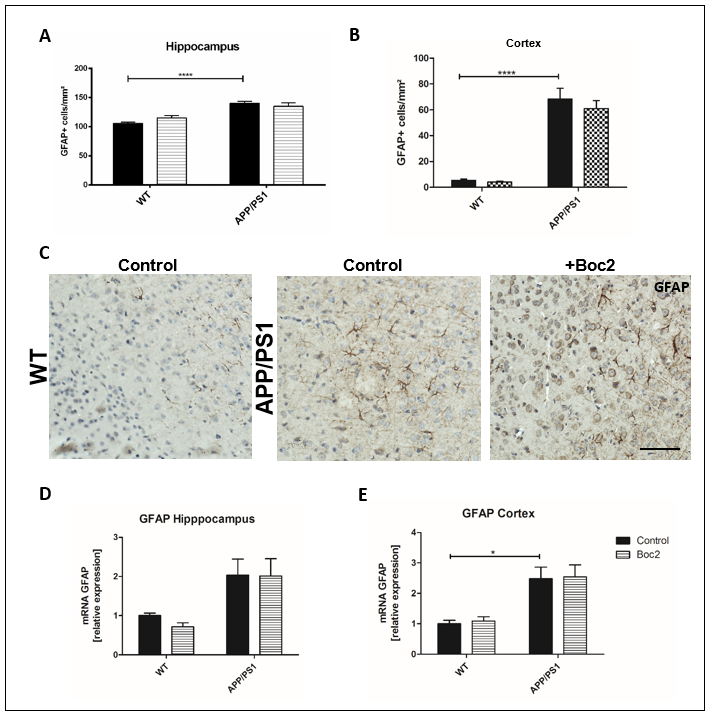


**Fig. S4 FPR modulation does not affect astrocytes in APP/PS1 mice A)** GFAP positive cells/mm² in the hippocampus where increased from WT control to APP/PS1 control mice.**B)** Also in the cortex we could see the same increased amount of GFAP positive cells in APP/PS1 control mice compared to WT control (n>15) **C)** Exemplary anti-GFAP staining’s of WT, APP/PS1 and APP/PS1+Boc2 mice in the cortex. **D)** Relative expression of *Gfap* mRNA in the hippocampus showed no differences but **E)** in the cortex we detected an increased *Gfap* mRNA expression in APP/PS1 control mice (n>6, ). *Scale bar* **c** 50 µm. Shown are the mean values of each group with SEM. Two-way ANOVA with turkey test *p <0.05 **p<0.01 ***p<0.001 ****p<0.0001

Table S1. Used primer pairs with sequences, specific annealing temperature and supplier information.

| **Name** | **Sequences (5’ -> 3’)** | **Annealing temp.** | **Supplier** |
| --- | --- | --- | --- |
| Itgam | QT00101145 | 55 °C | Qiagen |
| GFAP | QT00156471 | 55 °C | Qiagen |
| IDE | for’ CCGGCCATCCAGAGAATAGAA (21)  rev’ ACGGTATTCCCGTTTGTCTTCA (22) | 57°C | Eurofins |
| NEP | for’ CAGCCTCAGCCGAAACTAC (19)  rev’ CACCGTCTCCATGTTGCAGT (20) | 57°C | Eurofins |
| 18s | for’ GAATAATGGAATAGGACCGCGG (22)  rev’ AAGAATTTCACCTCTAGCGGCG (22) | 57°C | Eurofins |
| NGF | for’ ACCACAGCCACAGACATCAAGG (22)  rev’ GGTTAGGACAACTCTCACCCAC (22) | 55°C | Eurofins |
| BDNF | for’ AAAGTCCCGGTATCCAAAGGC (21)  rev’ TAGTTCGGCATTGCGGAGTTCC (22) | 57°C | Eurofins |
| TrkB | for’ CGCCCTGTGAGCTGAACTCTG (21)  rev’ CTGCTTCTCAGCTGCCTGACC (21) | 60°C | Eurofins |
| GDNF | for’ GGGCCTGAGGTCTATTACATC (21)  rev’ GTTTCTGAGGGCACGAAGGAG (21) | 58°C | Eurofins |
| TBP | for’ AGAACAATCCAGACTAGCAGCA (21)  rev’ GGGAACTTCACATCACAGCTC (21) | 59.5°C | Eurofins |
| RPL13A | for’ GGGCAGGTTCTGGTATTGGAT (21)  rev’ GGCTCGGAAATGGTAGGGG (19) | 60.5°C | Eurofins |
